# Supplementary material for: Controlling for openness in the male-dominated collaborative networks of the global film industry
Source: PLoS One. 2020 Jun 12;15(6):e0234460. doi: 10.1371/journal.pone.0234460 (PMC7292392; doi:10.1371/journal.pone.0234460)
Supplement: Attachment — Submitted filename: PlosOneRebuttalLetter.docx [file pone.0234460.s001.docx]

Joerg Heber,

Editor-in-Chief

*PLOS ONE*

April 7, 2020

Dear Dr. Heber,

I am pleased to provide a revised research article entitled “Controlling for openness in the male-dominated collaborative networks of the global film industry” by Deb Verhoeven, Katarzyna Musial, Stuart Palmer, Sarah Taylor, Shaukat Abidi, Vejune Zemaityte and Lachlan Simpson for consideration for publication in *PLOS ONE*. We are grateful for the Reviewer comments provided to us which have strengthened the article considerably. A summary of our consequent responses and revisions is:

| Review comment | Response |
| --- | --- |
| * Revise the title which currently is misleading | Removed the word “optimal” |
| * Clarify the new contributions from the current manuscript, particularly in the abstract | To emphasize the contributions of our work, we included in the abstract the following statement: “applying network analysis to understand inequality in the film industry has been undertaken. However, in this study we offer a comprehensive approach that enables us to not only understand what inequality in film industry looks like through the lens of network science but also how we can attempt to address this issue. We offer a data-driven simulation framework that investigates various what-if scenarios when it comes to network evolution. We then assess each of these scenarios with respect to its potential to address gender inequality in film industry.” |
| * Provide more information about the collection of the data and try to make some version of the data available | More detailed information is now provided via correctly detailed references |
| The manuscript indicates data curation and collection procedures can be found at the kinomatics website, but the linked reference leads to the general webpage and not the specifics of this study. In the manuscript, please include additional information about which data were collected for the study and what methods of collection were employed. | Have updated the URL in the footnotes to point to the descriptive pages |
| We note that you have indicated that data from this study are available upon request. PLOS only allows data to be available upon request if there are legal or ethical restrictions on sharing data publicly. | Have created DOIs for the data in Figshare: https//doi.org/10.6084/m9.figshare.11959221.v1 |
| In the abstract, the authors state that "we suggest that a study of network relationships offers innovative recommendations for understanding and redressing inequality in these industries.". I find a chance that this statement might be misleading with respect to what is done in the paper. It could be interpreted that the current paper is the first one to attempt a network approach; however, later (line 177) the authors write that "Lutter, who argues that the critical index of equity outcomes is network openness" showing that others have suggested similar things before.  Furthermore, the following statement "Inequality is exacerbated when industry networks are most closed" suggests that it is a finding of the current paper; as I understand it is also a known result from Lutter.  Clarifying the new contributions from the current manuscript, particularly in the abstract, would sharpen the manuscript and makes it easier for the reader to judge the importance of the paper. | To avoid the confusion we rephrased the sentence “Instead, we suggest that a study of network relationships offers innovative recommendations for understanding and redressing inequality in these industries.” to “Network science offers an alternative way of identifying and proposing change mechanisms, as it puts emphasis on relationships instead of individuals. Preliminary work on applying network analysis to understand inequality in the film industry has been undertaken.”  Also, we changed the following sentence “Inequality is exacerbated when industry networks are most closed.” Into “As suggested by previous studies, inequality is exacerbated when industry networks are most closed.” to make clear that it is not our finding.  To emphasize the contributions of our work, we included in the abstract the following statement: “However, in this study we offer a comprehensive approach that enables us to not only understand what inequality in film industry looks like through the lens of network science but also how we can attempt to address this issue. We offer a data-driven simulation framework that investigates various what-if scenarios when it comes to network evolution. We then assess each of these scenarios with respect to its potential to address gender inequality in film industry.” |
| I am slightly skeptical towards the formalization of Lutter's network openness in terms of small world (clustering coefficient, average shortest path), modularity, and assortativity. While these measures are common in studies of networks (and many networks are small-world-like and modular and assortative) we lack a proof of the external validity of these measures in the same way as demonstrated by Lutter. I am aware that these would probably require additional experiments which are beyond the scope of the current study. Thus, mentioning such limitations would make it easier to identify the open problems. | We would like to note that, in this study, we combined two approaches: (i) one for addressing inequality (Lutter’s network openness) and (ii) one for building good network of collaborations (small-world phenomenon). For Lutter’s openness we suggest to decrease the modularity and assortativity and for building healthy collaborative networks to promote small-world behaviour where friend-of-my-friend is my friend. We agree that there is lack of proof of the external validity of these measures in the same way as demonstrated by Lutter. To make it clear in the text we added the following to the section “Shortcomings of the approach”: “In an attempt to represent network openness in terms of network science, we propose to combine two approaches: (i) one for addressing inequality as a type of network marginalisation (Lutter’s network openness) and (ii) a second for building a good network of collaborations (small-world phenomenon). To achieve Lutter’s recommendation for network openness we suggest decreasing modularity and assortativity. For building a healthy collaborative network we suggest promoting small-world behaviour where friend-of-my-friend is my friend. The limitation resulting from our approach is a lack of proof of the external validity of these measures in the same way as demonstrated by Lutter and this will be one of the future directions for this study.” |
| How is the openness of the networks measured? In the abstract, it is stated that "the most critical factor for improving network openness is ...". From the paper, I understood that (line 210) "we define an open network as one that follows a small-world model and at the same time aims at reducing modularity and node-degree assortativity." The interventions decrease the latter two. However, they also decrease the clustering coefficient, which makes it less small-world and thus less open. In view of the lack of a (more) clear definition of network openness, I find the conclusion not quantitatively supported by the analysis. The title ("optimal openness") is misleading as the relation between the intervention and openness is qualitative. | We are aware that we did not quantify this and that this is a very challenging, if not impossible, task. We mentioned in the paper that: “There is a very thin line and natural trade-off between all these conditions. A small-world is characterized, among other factors, by a high clustering coefficient, but, at the same time, we claim that too high modularity (division into isolated groups) is not desirable. This we counter-balance by proposing a short average shortest path resulting from a small-world model.” This trade-off has to be carefully managed as we want to decrease modularity to split the men-only cliques identified in the data and in the same time keep the small-world character of the network. Decreasing modularity also decreases the clustering coefficient. However, smaller clustering coefficient does not mean that this network is not small-world anymore. We assure the small-world phenomenon by adding additional connections according to the friend-of-a-friend phenomenon, as proposed in FoF v1 and FoF v2. From how the clustering coefficient is defined (number of triples connected to a given node divided by the number of all possible triples connected to this node), when we start adding edges bridging women and men will cause that initially the clustering coefficient decreases but more parts of the networks will be connected. This will result in building bridges between isolated groups and, as a result, enhance the small-world phenomenon. To make clear the reasons why certain measures behave in certain way and to strengthen our argument we added this discussion to “Analysis and discussion” section.  We also, changed the title and removed “optimal”. We agree with the reviewer that “optimal” suggests that openness is in a form of formula. |
| I was hoping to find more information about the collection of the data (page 10), for example what sources/films/genres were included or excluded and for what reasons. This would be interesting to assess whether the differences in the network characteristics (Table 2) could be due to the choices made in the data collection/pre-processing. Regarding the sharing of the data: I understand that the underlying data is sensitive and that it cannot be shared. However, in order to reproduce the analysis (and build upon it), would it be possible to publish partial or anonymized datasets, for example simply the network and the gender of the node (without any further meta information)? | Network Data is now accessible. Have updated reference 38 to include precise URL link that describes the data collection process. |

Thank you for your consideration!

Sincerely,

Deb Verhoeven

Canada 150 Research Chair in Gender and Cultural Informatics

Department of Interdiscplinary Studies

University of Alberta
